# Hyperbaric oxygen preconditioning attenuates brain injury after intracerebral hemorrhage by regulating microglia polarization in rats

**DOI:** 10.1111/cns.13208

**Published:** 2019-08-14

**Authors:** Ming Wang, Lin Cheng, Zhong‐Liang Chen, Rajneesh Mungur, Shan‐Hu Xu, Jiong Wu, Xiao‐Li Liu, Shu Wan

**Affiliations:** ^1^ Department of Neurosurgery, College of Medicine, The First Affiliated Hospital Zhejiang University Hangzhou China; ^2^ Brain Center Zhejiang Hospital Hangzhou China

**Keywords:** hyperbaric oxygen precondition, inflammation, intracerebral hemorrhage, microglia

## Abstract

**Aims:**

Hyperbaric oxygen preconditioning (HBOP) attenuates brain edema, microglia activation, and inflammation after intracerebral hemorrhage (ICH). In this present study, we investigated the role of HBOP in ICH‐induced microglia polarization and the potential involved signal pathway.

**Methods:**

Male Sprague‐Dawley rats were divided into three groups: SHAM, ICH, and ICH + HBOP group. Before surgery, rats in SHAM and HBOP groups received HBO for 5 days. Rats in SHAM group received needle injection, while rats in ICH and ICH + HBOP groups received 100 μL autologous blood injection into the right basal ganglia. Rats were euthanized at 24 hours after ICH, and the brains were removed for immunohistochemistry and Western blotting. Neurological deficits and brain water content were determined.

**Results:**

Intracerebral hemorrhage induced brain edema, which was significantly lower in the HBOP group. The levels of MMP9 were also less in the HBOP group. HBO pretreatment resulted in less neuronal death and neurological deficits after ICH. Their immunoactivity and protein levels of M1 markers were downregulated, but the M2 markers were unchanged by HBOP. In addition, ICH‐induced pro‐inflammatory cytokine (TNF‐α and IL‐1β) levels and the phosphorylation of JNK and STAT1 were also lower in the HBOP rats.

**Conclusions:**

HBO pretreatment attenuated ICH‐induced brain injuries and MMP9 upregulation, which may through the inhibiting of M1 polarization of microglia and inflammatory signal pathways after ICH.

## 1 INTRODUCTION

Intracerebral hemorrhage (ICH), a devastating stroke subtype, is characterized by high mortality and disability without an effective treatment. Hyperbaric oxygen preconditioning (HBOP) provides neuroprotective effects in ischemia/hypoxia, trauma, and spinal cord injury.^1^, ^2^, ^3^, ^4^ Increasing evidences suggest that HBOP also plays beneficial role in ICH.^5^, ^6^

Studies suggest that inflammatory reaction is an important mechanism of secondary injury after ICH, which is the critical factor of high mortality and disability after ICH.^7^, ^8^, ^9^ Previous study demonstrated that HBOP could attenuate neuroinflammation after ICH.^10^ Microglia, the primary innate immune effector cells of the central nervous systems (CNS), plays a key role in the initial inflammatory response.^11^ Once activated, microglia serves as a double‐edged sword during the pathological process of disease in CNS. In response to acute brain injury, classically activated microglia (M1 phenotype) release devastating pro‐inflammatory cytokines and exacerbate inflammatory damage. On the other hand, alternative activated microglia (M2 phenotype) provide neuroprotective effects by phagocytosis and removal of cell debris, attenuate local inflammation, and tissue remodeling.^11^ Therefore, it is important to identify the status of polarized microglia and understand the regulation mechanism for future stroke management.^12^ Our previous study suggested that microglia was activated and polarized after ICH. M1 pheotypic microglia increased and peaked as early as 4 hours, remained at a high level (elevated) at day 3, and decreased at day 7 after ICH. M2 phenotypic microglia increased and peaked at day 1, and declined at day 7 after ICH.^13^

Based on previous study, we hypothesized that HBOP plays a role in microglia polarization and secondary inflammation after ICH.

## 2 METHODS

### 2.1 Experimental group

Rats were randomly divided into three groups: the SHAM group (sham operation after 5 days HBO pretreatment, 100% oxygen at 3ATA, 60 minutes per day); the ICH group (100 μL autologous blood injection in normobaric air); and the ICH + HBOP group (ICH operation after 5 days HBOP treatment). Rats were euthanized at 24 hours after ICH (n = 10 per group). All rats had behavioral tests before and 1 day after surgery.

### 2.2 HBO treatment

HBO‐treated rats were placed in a small rodent HBO chamber (Marine Dynamics Corp.). These rats were pressurized over 15 minutes to a plateau pressure of 3 atmosphere absolute (ATA) with 100% oxygen supplied continuously and maintained for 60 minutes. The temperature in chamber was maintained between 22°C and 25°C. Decompression was then carried out over 25‐30 minutes.

### 2.3 Intracerebral blood injection

Male Sprague‐Dawley rats (weighing 280‐320 g) were anesthetized with pentobarbital (40 mg/kg) intraperitoneally. Rectal temperature was maintained at 37.5°C by a feedback‐controlled heating pad. The right femoral artery was catheterized for blood collection, continuous blood pressure, and blood gas monitoring. The rats were positioned in a stereotactic frame and received an injection of 100 µL autologous blood into the right basal ganglia (coordinates 0.2 mm anterior, 5.5 mm ventral, 3.5 mm lateral to the bregma)^14^ at a rate of 10 µL/min with a 26‐gauge needle. After injection, the needle was kept for 5 minutes and then removed. The bone hole was sealed with bone wax, and subsequently, the skin incision was closed with a suture.

### 2.4 Brain water content measurement

Rats were anesthetized with pentobarbital (40 mg/kg) intraperitoneally (n = 6, per group). Brain was removed and divided into five parts: ipsilateral basal ganglia (Ipsi‐BG), contralateral basal ganglia (Contra‐BG), ipsilateral cortex (Ipsi‐cortex), contralateral cortex (Contra‐cortex), and cerebellum. Brain samples were immediately weighed on an electric analytic balance to obtain the wet weight and then dried at 100°C for 24 hours to obtain the dry weight. Brain water content was calculated using the following formula: brain water content (%) = (wet weight – dry weight)/wet weight × 100%.^15^

### 2.5 Brain histology and immunohistochemistry

Rats were anesthetized and perfused transcardially with 4% paraformaldehyde (PFA). Brains were removed and fixed with 4% PFA, dehydrated with 30% glucose, and then sectioned coronally (18‐μm‐thick slices). Immunohistochemistry and immunofluorescence staining were performed as described previously.^16^ The primary antibodies include rabbit anticluster of differentiation 16 (CD16) (1:200, Abcam), mouse anti‐inducible nitric oxide synthase (iNOS) (1:200, Abcam), rabbit antichitinase 3 like protein 3 (YM‐1) (1:400, Abcam), and rabbit anticluster of differentiation 206 (CD206) (1:200, Abcam).

### 2.6 Fluoro‐Jade C staining

Flouro‐Jade C staining was performed to assess neuronal degeneration, as described previously.^17^ Briefly, slides were dried on a slide warmer at 50°C for 30 minutes and treated with 0.06% potassium permanganate for 15 minutes. After a brief rinse in double‐distilled water, the sections were stained with 0.001% Fluoro‐Jade C in 1% acetic acid for 25 minutes on shaker in the dark. Then, sections were rinsed for 1 minute in double‐distilled water three times and followed by air‐drying on the slide warmer at 50°C for 10 minutes. Slides were then cleared in xylene and mounted.

### 2.7 Cell counting

Three different areas were selected in Ipsi‐BG around the hematoma in high‐power images (×40 magnification) for counting by a blinded investigator and expressed as cells per square millimeter. All measurements were repeated three times using an image analysis system (ImageJ) and carried out by a blinded investigator.

### 2.8 Western blot analysis

Western blot analysis was performed as previously described.^18^ Ipsi‐BG and Contra‐BG tissues were sampled. Briefly, protein concentration was determined by Bio‐Rad Protein Assay Kit. 50 μg protein sampled tissue was suspended in loading buffer, loaded on an SDS‐PAGE gel, and transferred onto a hybond‐C pure nitrocellulose membrane (Amersham, Pittsburgh, PA, USA). The membrane was blocked in Carnation nonfat mike for 1 hour and incubated with primary and secondary antibodies. The primary antibodies were goat antimatrix metallopeptidase 9 (MMP9) (1:1000, R&D), rabbit antidopamine‐ and cAMP‐regulated phosphoprotein, rabbit anti‐CD16 (1:1000, Abcam), mouse anti‐iNOS (1:1000, Abcam), rabbit anti‐YM‐1 (1:1000, Abcam), rabbit anti‐CD206 (1:1000, Abcam), rabbit anti‐TNF‐α (1:1000, Abcam), rabbit anti‐IL‐1β (1:1000, Abcam), rabbit anti‐TGF‐β (1:1000, Abcam), rabbit anti‐IL‐10 (1:1000, Abcam), rabbit anti‐JNK (1:1000, Cell Signaling Technology), rabbit anti‐P‐JNK (1:1000, Cell Signaling Technology), rabbit anti‐STAT1 (1:1000, Cell Signaling Technology), rabbit anti‐P‐STAT1 (Tyr701) (1:1000, Cell Signaling Technology), rabbit anti‐STAT3 (1:1000, Cell Signaling Technology), rabbit anti‐P‐STAT3 (Tyr705) (1:1000, Cell Signaling Technology), and mouse antiglyceraldehyde 3 phosphate dehydrogenase (GAPDH) (1:500 000, Cell signaling). The secondary antibody includes goat antirabbit, rabbit antigoat, and goat antimouse IgG (1:2000, Bio‐Rad). The antigen‐antibody complexes were visualized with the ECL chemiluminescence system (Amersham) and exposed to Kodak XOMAT film. The relative densities of bands were analyzed with ImageJ.

### 2.9 Behavioral tests

Two behavioral tests were used: corner turn and forelimb use asymmetry test.^19^ All animals were tested before and 1 day after surgery. These tests were performed by a blinded investigator.

### 2.10 Statistical analysis

All data were presented as mean ± SD and were analyzed by Student's *t* test for single comparisons or ANOVA for multiple comparisons as appropriate. Statistical significance was set at *P *< .05.

## 3 RESULTS

### 3.1 HBOP attenuated ICH‐mediated brain edema and the expression of MMP9

**Figure 1:**
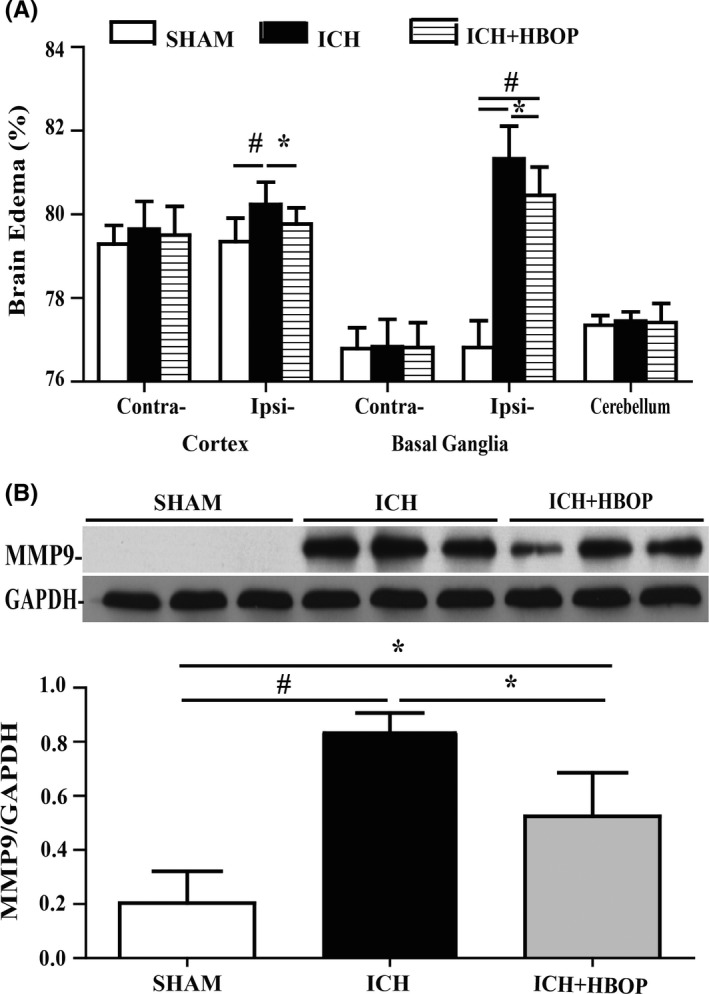
Effects of HBOP on brain edema and MMP9s at day 1 after ICH. A, Brain edema at day 1 after ICH in the sham, ICH, and HBOP groups. Values are mean ± SD, n = 6, per group, *P < .05, #*P *< .01. B, Western blot analysis of MMP9 protein lever in the perihematomal tissue at day 1 after ICH. Values are mean ± SD, n = 3, per group, *P < .05, #*P *< .01

The brain water contents in the ipsilateral cortex and basal ganglia were significantly higher in the ICH group compared with SHAM group at 24 hours (*P *< .01). HBO pretreatment attenuated ICH‐induced brain edema in ipsilateral cortex and basal ganglia (BG) (79.35% ± 0.39% vs 80.24% ± 0.53%, *P *< .05 in ipsilateral cortex, 80.46% ± 0.67% vs 81.33% ± 0.78%, *P *< .05 in ipsilateral BG, n = 6, Figure 1A).

Western blot analysis showed the MMP‐9 protein levels in the ICH + HBOP group were significantly lower than that in the ICH group (0.524% ± 0.093% vs 0.832% ± 0.043%, n = 3, *P *< .05, Figure 1B) at 24 hours after ICH.

### 3.2 ICH‐induced neuronal death and neurological deficits were reduced by HBOP

**Figure 2:**
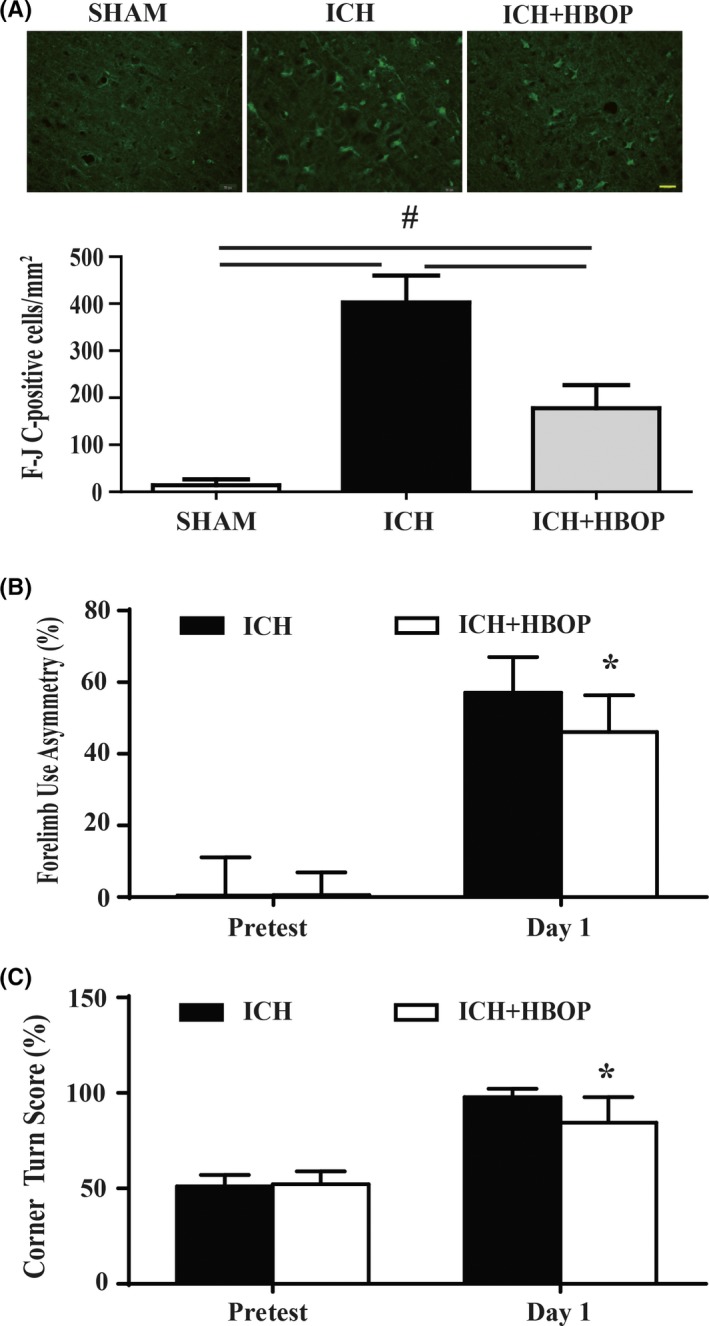
Effects of HBOP on neuronal protection and functional recovery after ICH. A, Fluoro‐Jade C‐positive cell counts the average of four locations in the perihematomal tissue at day 1 after ICH. Scale bar = 20 μm. Values are mean ± SD, n = 6, per group, #*P *< .01. B, Forelimb use asymmetry scores and C, corner turn scores at pretest and day 1 after ICH. Values are mean ± SD, n = 9, per group *P < .05

Neuronal degeneration was examined by Fluoro‐Jade C (F‐JC) staining. The number of the F‐JC‐positive cells and degenerating neurons were lower in ICH + HBOP group (177.8 ± 49.1/mm^2^) compared to ICH group (402.8 ± 57.2/mm^2^) at 24 hours after ICH, *P *< .01 (Figure 2A).

Hyperbaric oxygen preconditioning‐treated ICH animals achieved significantly better scores in forelimb use asymmetry test (46.1 ± 10.2 vs 57.1 ± 9.9, n = 9, *P *< .05, Figure 2B) and corner turn test (84.4 ± 13.3 vs 97.8 ± 4.4, n = 9, *P *< .05, Figure 2C) at 24 hours after ICH.

### 3.3 There were less M1, but not M2 polarization in HBOP animals after ICH

**Figure 3:**
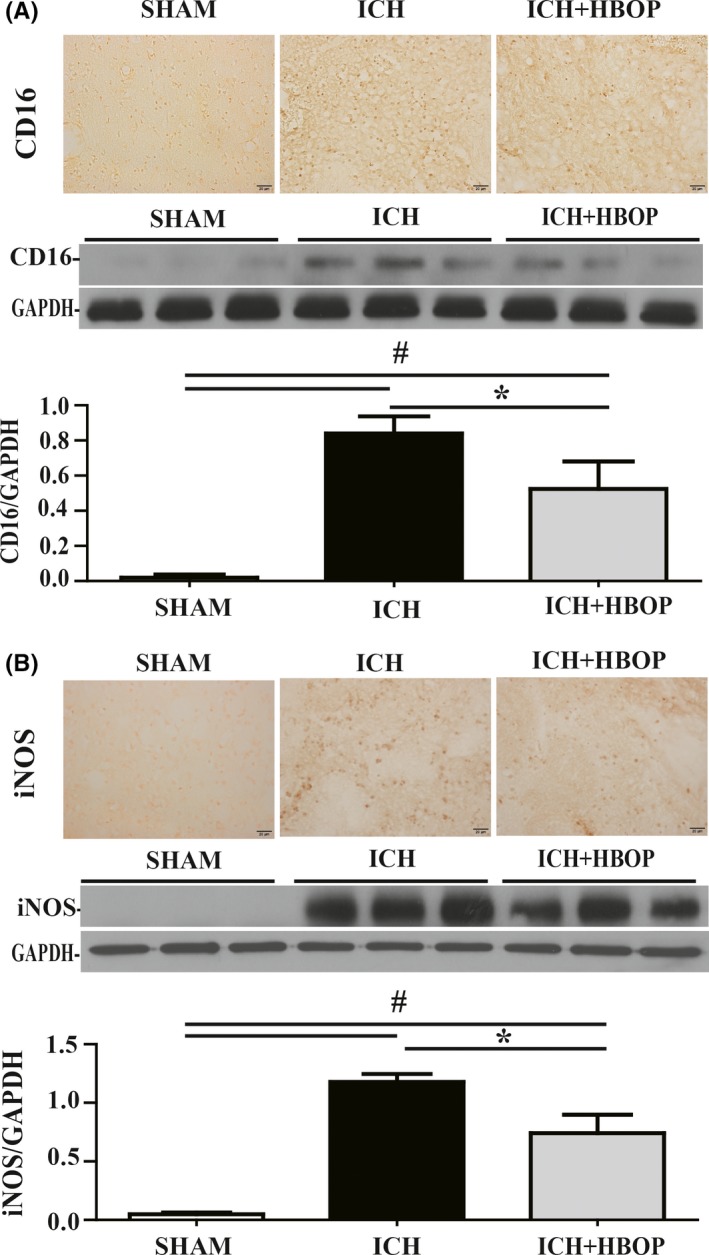
Effects of HBOP on M1 phenotypic microglia polarization at day 1 after ICH. Immunohistochemistry and Western blot analysis of CD16 (A) and iNOS (B) in the perihematomal tissue at day 1 after ICH. Values are mean ± SD, n = 3, per group in immunohistochemistry, n = 3, per group in Western blot analysis, *P < .05, #*P *< .01

Immunohistochemistry demonstrated that expression of CD16 and iNOS (markers of M1 phenotypic microglia/macrophage) was increased in both ICH and ICH + HBOP group, but there was less increase in HBOP rats compared with ICH group. Western blot analysis confirmed CD16 and iNOS protein levels were upregulated in the ipsilateral BG in both ICH and ICH + HBOP group, compared to SHAM group, but the levels were significantly lower in HBOP group (CD16/GAPDH 0.524 ± 0.090 in ICH + HBOP group vs 0.840 ± 0.057 in ICH group, n = 3, *P *< .05, Figure 3A, iNOS/GAPDH 0.741 ± 0.090 in ICH + HBOP group vs 1.178 ± 0.039 in ICH group, n = 3, *P *< .05, Figure 3B).

**Figure 4:**
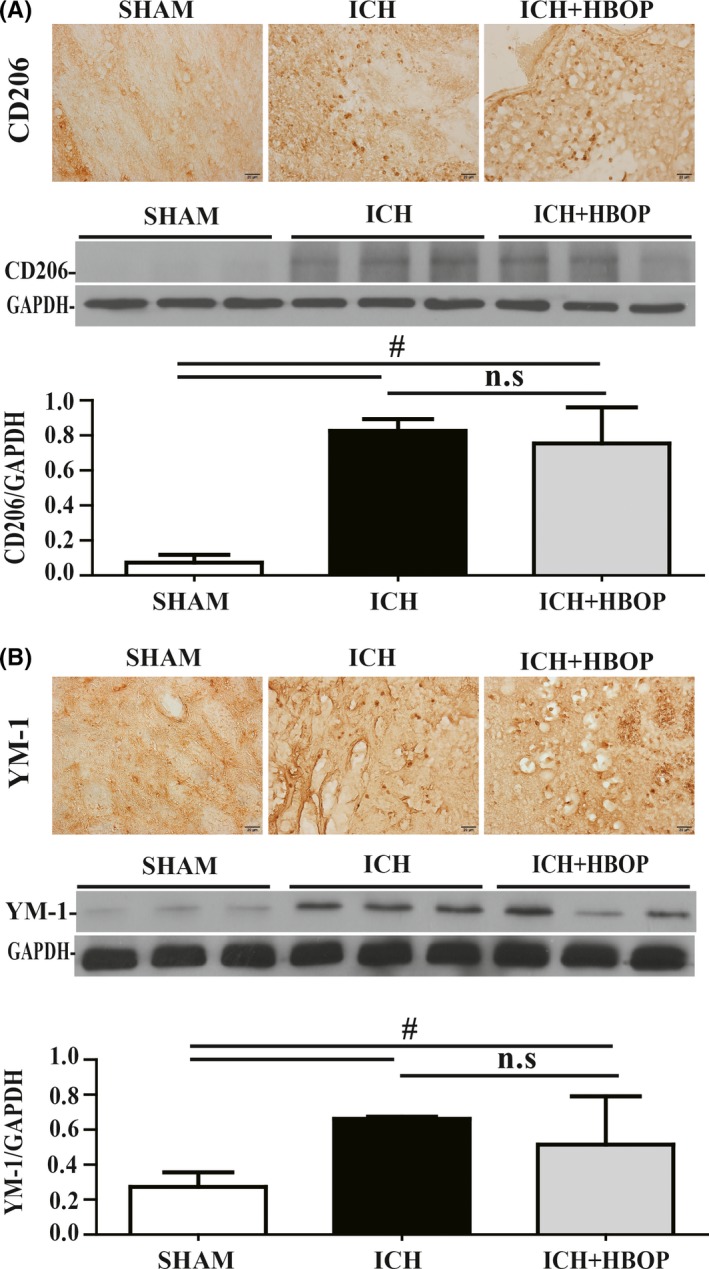
Effects of HBOP on M2 phenotypic microglia polarization at day 1 after ICH. Immunohistochemistry and Western blot analysis of CD206 (A) and YM‐1 (B) in the perihematomal tissue at day 1 after ICH. Values are mean ± SD, n = 3, per group in immunohistochemistry, n = 3, per group in Western blot analysis, *P < .05, #*P *< .01

Immunohistochemistry and Western blot analysis showed that the immunoreactivity and proteins levels of CD 206 and YM‐1 (markers of M2 phenotypic microglia/macrophage) were also increased after ICH, and there was no significant difference between ICH and ICH + HBOP group (CD206/GAPDH 0.753 ± 0.120 in ICH + HBOP group vs 0.826 ± 0.039 in ICH group, n = 3, *P *> .05, Figure 4A, YM‐1/GAPDH 0514 ± 0.159 in ICH + HBOP group vs 0.662 ± 0.007 in ICH group, n = 3, *P *> .05, Figure 4B).

### 3.4 HBOP reduces ICH‐induced pro‐inflammatory, but not anti‐inflammatory cytokines upregulation

**Figure 5:**
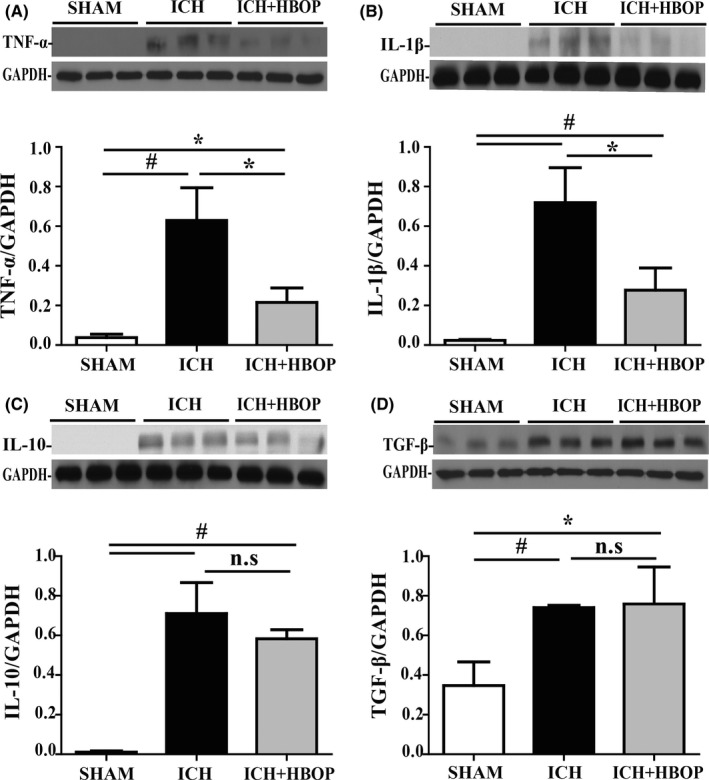
Effect of HBOP on inflammatory cytokines expression at day 1 after ICH. Western blot analysis of TNF‐α (A), IL‐1β (B), IL‐10 (C), and TGF‐ β (D) protein lever in the perihematomal tissue at day 1 after ICH. Values are mean ± SD, n = 3, per group, *P < .05, #*P *< .01

Western blot analysis demonstrated that ICH upregulated TNF‐α, IL‐1β (pro‐inflammatory cytokines), IL‐10, and TGF‐β (anti‐inflammatory) protein levels in the ipsilateral BG at 24 hours after ICG. The upregulation of TNF‐α and IL‐1β was significantly lower in HBOP group compared with ICH group (TNF‐α/GAPDH 0.216 ± 0.042 in ICH + HBOP group vs 0.630 ± 0.095 in ICH group, n = 3, *P *< .05, Figure 5A; IL‐1β/GAPDH 0.277 ± 0.065 in ICH + HBOP group vs 0.720 ± 0.101 in ICH group, n = 3, *P *< .05, Figure 5B). But there was no significant difference of IL‐10 and TGF‐β between ICH and ICH + HBOP group (IL‐10/GAPDH 0.583 ± 0.027 in ICH + HBOP group vs 0.710 ± 0.090 in ICH group, n = 3, *P *> .05, Figure 5C; TGF‐β/GAPDH 0.759 ± 0.108 in ICH + HBOP group vs 0.740 ± 0.007 in ICH group, n = 3, *P *> .05, Figure 5D).

### 3.5 HBOP inhibits ICH‐induced JNK/STAT1, but not STAT3 activation

**Figure 6:**
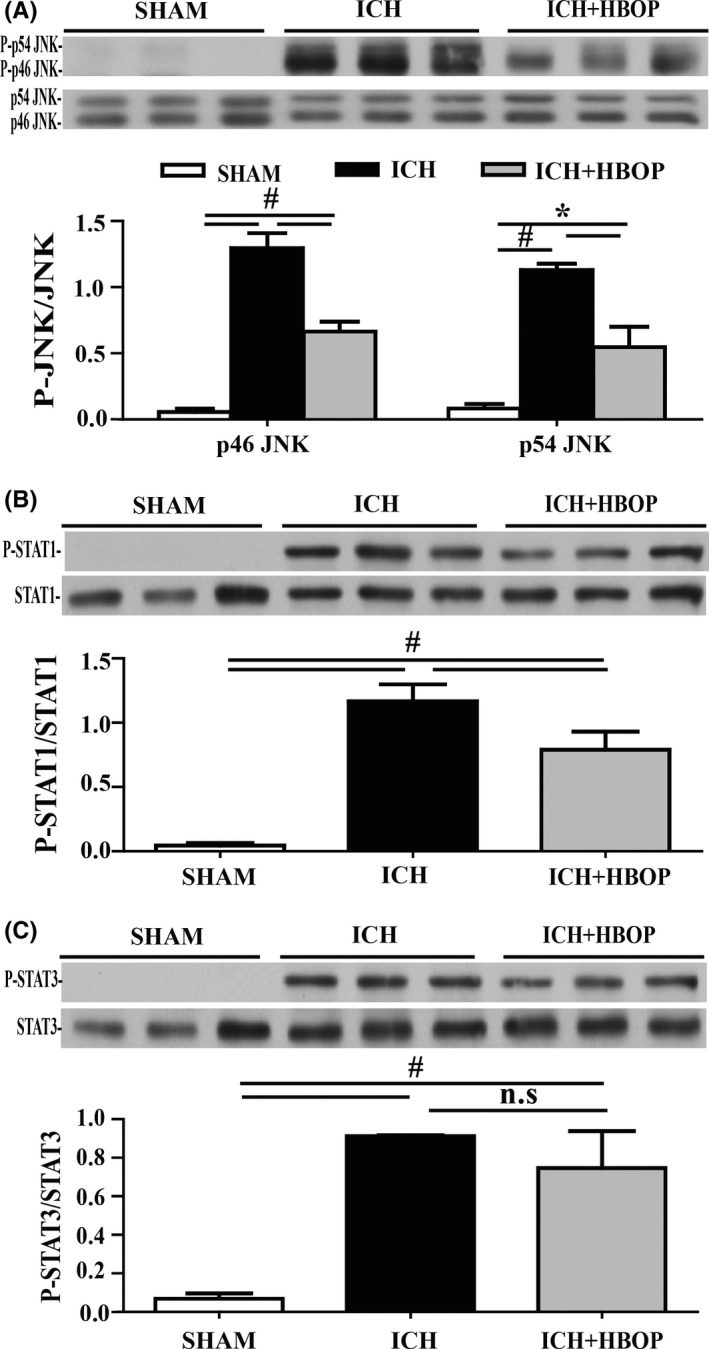
Effect of HBOP on JNK/STAT signal pathway activation at day 1 after ICH. Western blot analysis of p46, p54 JNK (A), STAT1 (B), and STAT3 (C) protein lever in the perihematomal tissue at day 1 after ICH. Values are mean ± SD, n = 3, per group, *P < .05, #*P *< .01

As we showed above, HBOP resulted in less M1 microglial polarization by reducing TNF‐α and IL‐1β levels. Furthermore, we tested the effect of HBOP on ICH‐induced activation of the major transcription factors involved in microglial cells activation and polarization. The levels of phosphorylated Jun N‐terminal kinase (P‐JNK) were significantly lower in HBOP rats (P‐p46 JNK/p46 JNK 0.66 ± 0.08 in ICH + HBOP group vs 1.30 ± 0.11 in ICH group, n = 3, *P *< .01; P‐p54 JNK/p54 JNK 0.54 ± 0.15 in ICH + HBOP group vs 1.13 ± 0.05 in ICH group, n = 3, *P *< .05, Figure 6A). Signal transducer and activator of transcription 1 (STAT1) and STAT3 are members of STAT families and downstream regulatory factors of the JNK pathway, involved in regulating different subtypes of microglia cells activation and polarization. HBO pretreatment downregulated the expression of phosphorylated STAT1 (P‐STAT1/STAT1 0.79 ± 0.08 in ICH + HBOP group vs 1.17 ± 0.8 in ICH group, n = 3, *P *< .01, Figure 6B), but has little effect on the expression of phosphorylated STAT3 (P‐STAT3/STAT3 0.75 ± 0.11 in ICH + HBOP group vs 0.91 ± 0.003 in ICH group, n = 3, *P* = .21, Figure 6C).

## 4 DISCUSSION

The major findings of this study are as follows: (a) HBOP attenuated ICH‐induced brain edema, neuronal death, and neurological deficits; (b) HBOP attenuated M1, but not M2 microglia/macrophage polarization, and reduced TNF‐α and IL‐1β levels; (c) the neuroprotective effect of HBOP may be regulating JNK/STAT pathway.

Studied indicated that HBOP has positive effects on traumatic brain injury,^1^, ^20^ ischemic stroke,^21^, ^22^ and spinal cord injury.^2^, ^3^ Recently, there is also discovered that HBOP provides neuroprotective effect on ICH.^6^, ^7^ HBOP‐induced activation of ribosomal protein S6 kinases (p70 S6 K), an important enzyme in protein synthesis, causes HO‐1 upregulation and reduces brain edema.^5^ Also, HBOP attenuated ICH‐induced brain edema by downregulation of aquaporin‐4 (AQP‐4) expression at days 1, 2, and 7 after ICH.^6^

Neuroinflammation is considered to be the most important mechanism of secondary brain injury after ICH.^8^, ^23^ Microglia play a key role in inflammation in the CNS.^12^, ^24^, ^25^ Increasing evidences indicate that activated microglia serve as a double‐edged sword during the pathological process of disease. In response to acute brain injury, M1 release devastating pro‐inflammatory cytokines and exacerbate inflammatory damage. On the other hand, M2 phenotypic microglia provide neuroprotective effects by phagocytosis, the removal of cell debris, reducing local inflammation, and improving tissue remodeling.^11^, ^13^, ^26^ Yang et al^10^ demonstrated that HBOP can reduce microglia/macrophage activation, TNF‐α expression, and neuronal death after ICH. The present study demonstrated that HBOP could inhibit M1 phenotypic microglia/macrophage activation and reduce the expression of pro‐inflammatory cytokines (TNF‐α and IL‐1β) after ICH, but had less effect on M2 phenotypic microglia/macrophage activation and expression of neurotropic cytokines (IL‐10 and TGF‐β). Additionally, the animals with HBOP had less expression of MMP‐9, an important regulator of blood‐brain barrier disruption after injury.^27^ Therefore, these animals had less brain edema, neuronal loss, and neurological deficits.

Also, we explored the mechanisms of HBOP on the regulation of microglia/macrophage polarization. Studies confirm lipopolysaccharide (LPS) induces inflammatory response in microglia/macrophage by activating several signaling pathways. Lipopolysaccharide first binds with membrane‐bound Toll‐like receptor 4 (TLR4), to activate its downstream signaling molecules, mainly mitogen‐activated protein kinases (MAPKs) and NF‐κB, through induction by phosphorylation.^28^, ^29^, ^30^ Phosphorylated MAPKs activate their downstream molecules, which can be translocated into the nucleus and contribute to expression of inflammatory molecules.^31^ MAPKs consist of the JNK, p38 MAPK, and p42/p44 MAPKs.^32^ STAT families are downstream regulator of JNK pathway and involved in sequential dimer formation, nuclear translocation, binding to specific DNA sequences, and regulating cell proliferation.^33^, ^34^ Studies indicated activation of STATs involved in microglia/macrophage polarization.^35^, ^36^, ^37^ STAT1 is related to M1 response, while STAT3 is related to M2 response, especially M2c subcategory, which is associated with tissue repair, extracellular matrix repair, and de‐activation of M1/Th1 immune response.^38^, ^39^, ^40^ Thus, we hypothesized whether HBOP regulates microglia/macrophage polarization through those signal pathways. Indeed, the present study confirmed that HBOP can down‐regulate the expression of P‐JNK and STAT1, reduce M1 polarization, and inhibit expression of pro‐inflammatory cytokines after ICH.

There are several limitations in this study: (a) using JNK/STAT inhibition to confirm the effect of this pathway was not performed in this study; (b) the effects of HBOP were only tested during acute phase after ICH. Long‐term brain tissue loss and behavioral deficits should be tested; (c) sex and age differences were not examined in this study.

## 5 CONCLUSIONS

Our results demonstrated that HBOP can attenuate ICH‐induced brain edema, neuronal loss, and neurological deficits. Hyperbaric oxygen preconditioning also attenuates M1 microglia/macrophage activation, reduces the expression of pro‐inflammatory cytokine (TNF‐α and IL‐1β), and MMP‐9. The effects of HBOP on ICH‐induced brain injury and microglia/macrophages may be through JNK/STAT pathway.

## CONFLICT OF INTEREST

The authors declare no conflict of interest.
